# Mechanical Strain Regulates Myofibroblast Differentiation of Human Scleral Fibroblasts by YAP

**DOI:** 10.3389/fphys.2021.712509

**Published:** 2021-09-30

**Authors:** Di Hu, Junhong Jiang, Baiyang Ding, Kang Xue, Xinghuai Sun, Shaohong Qian

**Affiliations:** ^1^Department of Ophthalmology & Visual Science, Eye & ENT Hospital, Shanghai Medical College, Fudan University, Shanghai, China; ^2^NHC Key Laboratory of Myopia, Chinese Academy of Medical Sciences, and Shanghai Key Laboratory of Visual Impairment and Restoration (Fudan University), Shanghai, China; ^3^Department of Ophthalmology, Children's Hospital of Fudan University, National Children's Medical Center, Shanghai, China; ^4^The Eye Hospital, School of Ophthalmology and Optometry, Wenzhou Medical University, Wenzhou, Zhejiang, China; ^5^Spine Research Center of Wannan Medical College, Wuhu, China; ^6^State Key Laboratory of Medical Neurobiology and MOE Frontiers Center for Brain Science, Institutes of Brain Science, Fudan University, Shanghai, China

**Keywords:** mechanical strain, sclera, myofibroblast differentiation, extracellular matrix remodeling, glaucoma

## Abstract

Scleral extracellular matrix (ECM) remodeling is thought to play a critical role in the pathogenesis of glaucoma. Mechanical strain induced by elevated intraocular pressure can promote myofibroblast differentiation of fibroblasts and result in scleral ECM remodeling; however, the underlying mechanism remains poorly understood. Yes-associated protein (YAP) is a mechanosensory protein and the key downstream transcriptional effector of the Hippo signaling pathway. Here, we investigated the role of YAP in mechanical strain-induced myofibroblast transformation during glaucoma scleral ECM remodeling. Integrative bioinformatics analyses were performed to identify the key pathways for the ECM remodeling of the sclera in glaucoma. Sprague–Dawley rats were used to establish a chronic ocular hypertension model, and the expression of collagen type I (COL1) and YAP in the sclera was analyzed by immunohistochemical analysis and Western blotting. Furthermore, human scleral fibroblasts (HSFs) were cultured and subjected to mechanical strain. In groups with or without the YAP siRNA or YAP inhibitor, cell proliferation, migration capacity, and the expression levels of YAP, COL1, and α-smooth muscle actin (α-SMA) were evaluated by Cell Counting Kit-8 assay, scratch assay, and Western blotting. The interactions between YAP and Smad3 were demonstrated by coimmunoprecipitation, and the expression levels of COL1 and α-SMA were evaluated in groups treated with or without the Smad3 inhibitor. We first revealed that the Hippo signaling pathway may be involved in mechanical strain-induced scleral ECM remodeling through bioinformatics analysis. Furthermore, the *in vivo* study showed upregulated YAP, COL1, and α-SMA expression in the hypertensive sclera of rats. *In vitro*, mechanical strain increased YAP and COL1 expression in HSFs and promoted myofibroblast differentiation. After YAP knockdown or inhibition with verteporfin, mechanical strain-induced fibrotic changes in HSFs were markedly suppressed. Additionally, YAP showed a protein interaction with Smad3, and the upregulation of a-SMA and COL1 in response to mechanical strain was also significantly downregulated following the inhibition of Smad3. In conclusion, mechanical strain activated scleral myofibroblast differentiation *via* YAP. The YAP pathway may play an important role in regulating scleral myofibroblast differentiation and ECM remodeling of the sclera in glaucoma.

## Introduction

Glaucoma is a group of multifactorial optic neuropathies characterized by progressive retinal ganglion cell (RGC) degeneration and corresponding visual field loss (Kwon et al., 2009). Although multiple factors contribute to its pathophysiological process, elevated intraocular pressure (IOP) plays an absolutely important role in the development and progression of glaucoma, and reducing IOP is currently the only effective therapy for glaucoma (The Advanced Glaucoma Intervention Study (AGIS), 2000; Anderson et al., 2003). The optic nerve head (ONH) is considered to be the primary site of damage in glaucoma (Quigley et al., 1981). The RGC axons converge at the ONH, pass through the laminar cribrosa (LC), enter the scleral canal, and exit the eye. IOP elevation induces mechanical stresses and strains within ONH cause the scleral canal opening to widen circumferentially and the LC to bow posteriorly, resulting in extension, compression, or shearing of RGC axons, then eventually contributing to glaucomatous optic neuropathy (Burgoyne et al., 2005; Sigal et al., 2007; Crawford Downs et al., 2011; Downs et al., 2011; Wang et al., 2017).

The peripapillary sclera (PPS) and LC are the main load-bearing connective tissues of the ONH. The IOP-related biomechanical response of the ONH is determined by the mechanical properties of the sclera, especially the adjacent PPS (Sigal, 2009; Nguyen et al., 2017). *Ex vivo* and computational modeling studies show that the magnitude of biomechanical strain within the LC depends strongly on the scleral stiffness, and increasing the PPS stiffness reduces the LC strain (Sigal et al., 2004; Coudrillier et al., 2016). An experimental study reported that stiffening the PPS ring with collagen cross-linking can reduce the IOP-related biomechanical sensitivity of the optic nerve and LC complex (Thornton et al., 2009). Furthermore, an animal study showed that eyes from mice with microfibril deficiency had increased susceptibility to IOP-induced optic nerve degeneration (Wu et al., 2020). These data suggest that the biomechanics of PPS play an important role in glaucomatous damage (Bellezza et al., 2003; Ivers et al., 2016).

Scleral fibroblasts and their extracellular matrix (ECM) are the key components of the sclera, and the biomechanical properties of PPS are largely dependent on the scleral ECM, which is composed of collagen fibrils, proteoglycans, and elastin (Boote et al., 2020). Fibroblasts are mechanosensitive cells that are the predominant producers of ECM (Baum and Duffy, 2011; Duffy, 2011). Mechanical strain induced by elevated IOP can activate scleral fibroblast differentiation into myofibroblasts, which are responsible for ECM synthesis, secretion, and remodeling (Qu et al., 2015; Bergmeier et al., 2018; DeLeon-Pennell et al., 2020). Myofibroblast differentiation of scleral fibroblasts is thought to play a critical role in mechanical strain-induced scleral ECM remodeling (Oglesby et al., 2016; Qiu et al., 2020). Biomechanical studies show that the ocular rigidity of glaucoma patients was greater than that of control eyes (Hommer et al., 2008; Ebneter et al., 2009), and the stiffening of PPS has also been observed in human and experimental monkey glaucoma (Quigley et al., 1991; Coudrillier et al., 2012). Animal studies have shown increased scleral stiffness in glaucomatous eyes of both monkeys (Downs et al., 2005; Girard et al., 2011) and mice (Nguyen et al., 2013). Stiffening of the sclera may be a protective response to IOP-induced mechanical strain within the ONH. Although several biomolecular factors, such as matrix metalloproteinases (MMPs) and tissue inhibitor of matrix metalloproteinases (TIMPs), have been identified as key mediators of scleral ECM remodeling (Shelton and Rada, 2007; Downs et al., 2011), how IOP-related mechanical strain on the sclera stimulates the differentiation of fibroblasts into myofibroblasts remains poorly understood.

Yes-associated protein (YAP) is the key downstream transcriptional effector of the Hippo signaling pathway which is a critical mechanosensitive signaling pathway (Wada et al., 2011; Codelia et al., 2014). The Hippo/YAP pathway modulates a variety of biological processes, namely, regulating cellular proliferation, survival, and differentiation (Dey and Varelas, 2020), and has been more recently associated with fibrogenesis (Byun et al., 2019; Yao et al., 2020). As a tissue mechanosensor, YAP can be activated by mechanical stress and promote and sustain fibrosis (Dupont et al., 2011; Szeto et al., 2016; Liang et al., 2017). During the development of fibrosis, activated YAP can promote fibroblast differentiation into myofibroblasts and induce ECM production (Liu et al., 2015; Gui et al., 2018); on the other hand, increased YAP expression facilitates myofibroblast formation and stimulates myofibroblast accumulation (Xu et al., 2016; Toyama et al., 2018). Furthermore, YAP and its transcription targets are important regulators of ECM remodeling in glaucoma (Zhang and Kong, 2020). We hypothesized that YAP may play an important role in mechanical strain-induced myofibroblast transformation during glaucoma scleral remodeling. In this study, we investigated whether YAP affects fibroblast-to-myofibroblast differentiation in a glaucoma model.

## Materials and Methods

All animal procedures were under the Association for Research in Vision and Ophthalmology Statement for the Use of Animals in Ophthalmic and Vision Research. The experimental protocols were approved by the Institutional Review Board and Ethics Committee of Eye and Ear, Nose, Throat Hospital of Fudan University.

### Bioinformatics Analysis

First, genes associated with glaucoma, sclera, mechanical strain, and ECM remodeling were determined by the text mining tool pubmed2ensembl (http://pubmed2ensembl.ls. manchester.ac.uk/) (Yu et al., 2010). To determine the genes most closely related to mechanical strain-induced scleral ECM remodeling during glaucoma, we further performed the Gene Ontology biological process and Kyoto Encyclopedia of Genes and Genomes (KEGG) pathway enrichment analysis for text mining genes by using GeneCodis (http://genecodis.cnb.csic.es/) (Nogales-Cadenas et al., 2009). Then, a protein-protein interaction (PPI) network was constructed by STRING database (https://string-db.org/cgi/input.pl) (von Mering et al., 2003) and module analysis was performed using molecular complex detection in Cytoscape software (Smoot et al., 2011). Finally, KEGG analyses of the significant gene modules were obtained using DAVID (Database of Annotation, Visualization and Integrated Discovery, https://david.ncifcrf.gov/) platform (Huang et al., 2007).

### Chronic Ocular Hypertension (OHT) Model

Sprague–Dawley (SD) rats were housed in standard cages with a 12-h light-dark cycle. Chronic ocular hypertension (OHT) models were established in 8-week-old SD rats weighing 180–200 g (males). In each rat, the IOP was elevated in one eye by injecting a 0.3% carbomer solution (Carbomer 940 polymer, Solarbio, Shanghai, China) into the anterior chamber injection, as described previously (Kim et al., 2013), leaving the contralateral eye as a normotensive control. A 33-gauge needle (Kindly, Shanghai, China) was used to puncture near the corneal limbus, and a 33-gauge syringe (Hamilton, Ghiroda, Romania) was then used to inject 20 μl of 0.3% carbomer solution into the anterior chamber. All injections were made under anesthesia, with an intraperitoneal injection of 10% chloral hydrate (3 ml/kg; Macklin, Shanghai, China). After the procedure, experimental eyes were treated with antibiotic ointment (ofloxacin; Shengyang Xingqi Pharmaceutical Co., Ltd, China). The injection was repeated once the IOP declined below the successful level, which was at least 5 mmHg above the IOP of the contralateral eyes (Chan et al., 2007).

Intraocular pressure (IOP) measurements were performed in both eyes before anterior chamber injection and two times a week after the models were made. IOP was measured by a rebound tonometer (TonoLab, ICare, Espoo, Finland) between 10:00 a.m. and 12:00 a.m. while the animals were under anesthesia. Measurements were repeated at least 10 times for each eye, and the average value was used. After the models were successfully established for 1 week, scleral tissues were collected (Qiu et al., 2020).

### Sclera Fibroblast Isolation and Culture

Methods for isolation and culture of scleral peripapillary fibroblasts were described previously in detail (Qiu et al., 2018). Briefly, donor eyes were obtained from the Eye Bank of Eye and Ear, Nose, Throat Hospital of Fudan University within 24 h postmortem. Consent was obtained for using the eyes for research purposes. According to the medical history, no donor had a history of ocular disease other than cataracts. The PPS, a 2-mm-wide circular scleral band surrounding the ONH, was carefully dissected from donor eyes and digested by collagenase NB4 (Serva, Heidelberg, Germany) overnight at 37°C. After filtration (70 μm; Falcon; BD, Franklin Lakes, NJ, USA) and resuspension, human scleral fibroblasts (HSFs) were cultured in Dulbecco's modified Eagle's medium (DMEM; Gibco, Grand Island, NY, USA) with 20% fetal bovine serum (FBS) and 1% penicillin-streptomycin (Hyclone, South Logan, UT, USA) at 37°C in 5% CO_2_. After 1 week, media were replaced every 3 days. HSFs after passage 1 were grown in DMEM with 15% FBS and 1% penicillin-streptomycin. All experiments were done on passage numbers 4–7.

### Mechanical Stimulation

The Flexcell FX-5000 Tension System (Flexcell International Corporation, Burlington, NC, USA) was used to produce mechanical cyclic stretch to the HSFs. HSFs were seeded on collagen I-bonded Bioflex six-well plates (Flexcell International Corporation, Burlington, North Carolina, USA) at a density of 2–5 × 10^5^ cells/well. After 24 h of culture with DMEM containing 10% FBS and 24 h of cultivation with serum-free DMEM, the cells were replenished with media containing 1% FBS before mechanical strain. HSFs were subjected to continuous mechanical force with a biaxial sinusoidal waveform with 10% elongation and a frequency of 0.5 Hz. Control cultures were grown under the same conditions but without the strain protocol.

### Coimmunoprecipitation (Co-IP) Assay

Fibroblasts were washed with phosphate-buffered saline (PBS) two times, resuspended in cold radioimmunoprecipitation assay (RIPA) lysis buffer (Thermo Fisher Scientific, Shanghai, China), and lysed on ice for 2 h at 4°C. After centrifuging at 12,000 rpm at 4°C for 15 min, the supernatant was immediately transferred to new tubes. The supernatants were incubated with primary antibody (YAP, 1:50 Cell Signaling Technology, Danvers, USA) and non-immune IgA at 4°C for 4 h. Protein A/G beads (60 μl, Thermo Scientific, Rockford, IL) were then added to the mixture and incubated at 4°C for 4 h. The immunoprecipitated proteins were washed by centrifugation at 1,000 rpm at 4°C two times in cold PBS, resuspended in 30 μl 1x serine dehydratase (SDS) loading buffer, and boiled at 100°C for 10 min. The sample was then subjected to Western blot analysis.

### Western Blotting

The proteins of scleral tissues or cultured fibroblasts were extracted in conventional RIPA buffer (Thermo Fisher Scientific, Shanghai, China). A total of 20 ug of protein was separated by sodium lauryl sulfate-polyacrylamide gel electrophoresis and then wet transferred to a nitrocellulose membrane. After blocking with 5% bovine serum albumin (BSA) for 1 h at room temperature, the blocking solution was removed and anticollagen type I (1:1,000, Novus Biologicals, CO, USA), α-SMA (1:1,000, Abcam, Cambridge, UK), YAP (1:1,000, Cell Signaling Technology, Danvers, USA), or GAPDH (1:3,000, Bioworld, MN, USA) was added to 5% BSA and incubated at 4°C overnight. After rinsing three times with Tris-buffered saline Tween 20 (TBST), secondary antibodies were added and incubated for 1 h at room temperature. The blot was then washed three times with TBST and exposed.

### Small Interfering RNA Transfection

When the cells reached 50% confluence, HSFs were transfected with human YAP siRNAs to knock down the expression of YAP. Transfection of siRNA was performed using liposome transfection reagent (Lipofectamine 3,000), and cells transfected with control siRNA served as the experimental control. The YAP siRNA sequences were as follows: CCGUUUCCCAGACUACCUUTT and AAGGUAGUCUGGGAAACGGTT. The negative control siRNA sequences were UUCUCCGAACGUGUCACGUTT and ACGUGACACGUUCGGAGAATT. YAP siRNA and control siRNA were designed and synthesized by GenePharma Biological Company (Shanghai, China). Subsequent experiments were performed after 24 h. The knockdown efficiency of the siRNAs was detected by Western blot.

### Immunohistochemistry

The sclera was cut into 4-μm-thick sections. Then, the sections were deparaffinized in xylene (Thermo Fisher Scientific, Fairlawn, NJ, USA) at room temperature for 45 min and gradually rehydrated in 100, 95, 85, and 75% ethanol. After blocking with 5% BSA, the sections were incubated with primary antibody and incubated for 1 h at 37 and 4°C overnight. After further washing in TBS, the sections were incubated with a secondary antibody for 1 h at 37°C with the appropriate secondary antibodies.

### Immunofluorescence

Scleral sections were fixed in 4% paraformaldehyde, permeabilized, and blocked with 0.3% Triton-X 100 and 5% donkey serum. Sections were incubated with the following primary antibodies: YAP (1:1,000, Cell Signaling Technology, Danvers, USA) and α-SMA (1:1,000, Abcam, Cambridge, UK) at 4°C overnight. The tissues were washed with PBS three times and then incubated with Alexa Fluor 488-conjugated goat antirabbit (1:500, Thermo Fisher Scientific, Waltham, USA), Alexa Fluor 594-conjugated goat antirabbit (1:500, Thermo Fisher Scientific, Waltham, USA) for 1 h. The nuclei were stained with 4′,6-diamidino-2-phenylindole. Fluorescence images were obtained using the Nikon Eclipse E800 microscope (Nikon, Melville, NY, USA). Immunofluorescence quantification was performed using ImageJ software (ImageJ 1.53, http://imagej.nih.gov/ij/, National Institutes of Health, Bethesda, MD, USA).

### Migration Assay

An *in vitro* scratch assay was used to assess the migration capacity of HSFs. The assay was performed as previously reported (Qiu et al., 2018). Briefly, cells were seeded into six-well cell culture plates at a density of 2.5 × 10^5^ cells/well. After 24 h of incubation, the cell monolayer was scratched with a sterile pipette tip. HSFs were subjected to 10% cyclic strain at 0.5 Hz for 8 h. The wound areas were photographed at 0 and 24 h after scratching using an inverted microscope (Laxco, Mill Creek, WA, USA). The relative migration ratio of the cells was calculated according to the formula: (width at 0 h—width at 24 h)/width at 0 h.

### Cell Proliferation Assay

A Cell Counting Kit-8 (CCK-8) (Yeason, Shanghai, China) assay was used to assess the proliferation of HSFs. Cells were seeded into six-well culture plates. The CCK-8 assay was performed as previously reported (Qiu et al., 2018). The absorbance values at a wavelength of 450 nm were measured using a microplate reader (BioTek; Synergy HTX, Winooski, VT, USA).

### Statistical Analysis

Each experiment was repeated in triplicate for each assay, and data are expressed as the mean ± standard deviation. Comparisons between groups were analyzed using a two-tailed Student's *t*-test and *P* < 0.05 was considered statistically significant. All data were analyzed using GraphPad Prism software version 8.4 (GraphPad Software, Inc., San Diego, CA, USA).

## Results

### The Hippo Signaling Pathway May Contribute to Mechanical Strain-Induced Scleral ECM Remodeling in Glaucoma

**Figure 1:**
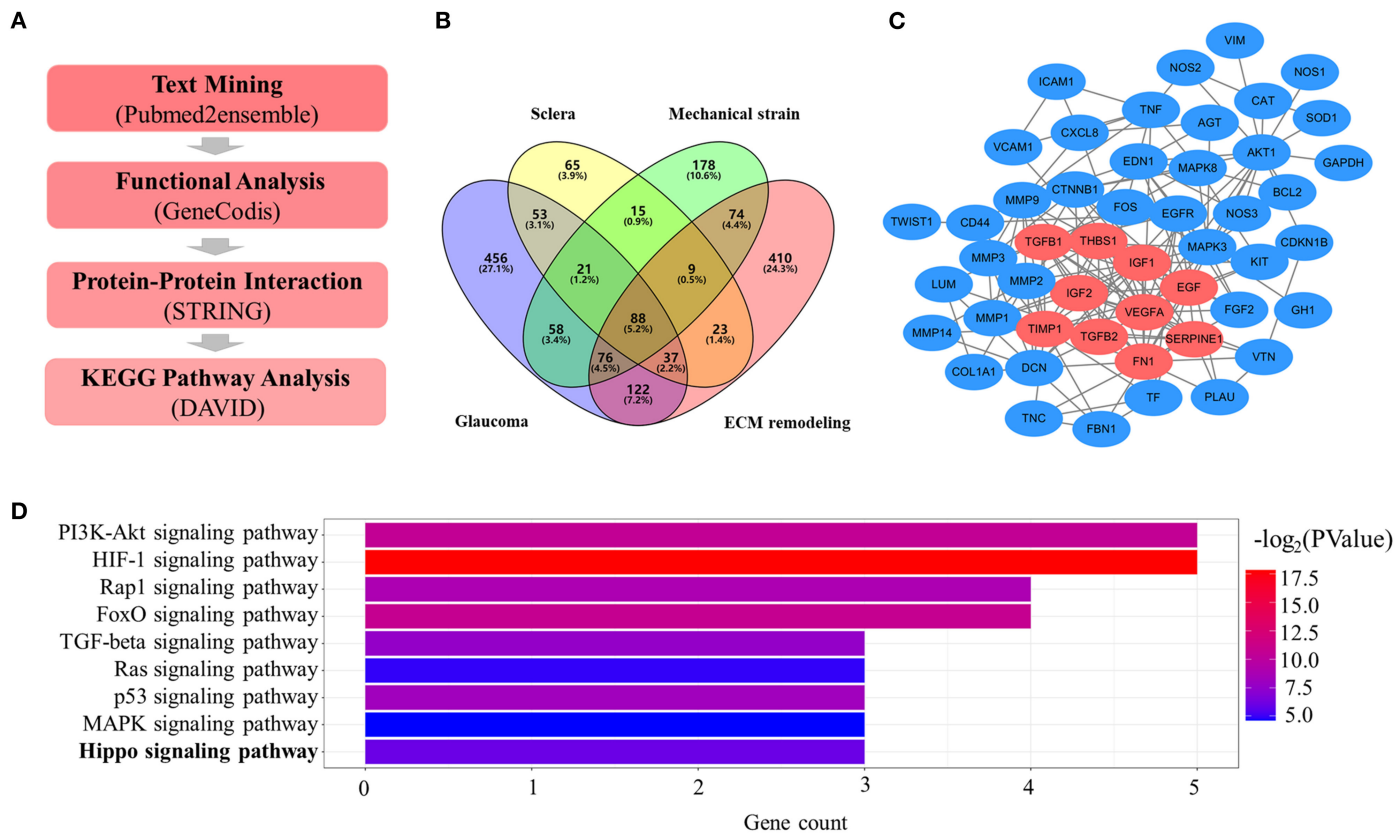
Key signaling pathway in mechanical strain-induced scleral ECM remodeling in glaucoma. **(A)** Overall bioinformatics analysis strategy. **(B)** Venn diagram of the text mining genes related to glaucoma, sclera, mechanical strain, and EMC remodeling. The 88 common genes were considered to be related to mechanical strain-induced scleral ECM remodeling in glaucoma. **(C)** The protein-protein interaction (PPI) network of the 54 target text mining genes, red circles represent the significant module genes. **(D)** Significantly enriched KEGG signaling pathways in gene module. Abbreviations: EMC, extracellular matrix; KEGG, Kyoto Encyclopedia of Genes and Genomes.

The overall bioinformatics analysis strategy is shown in Figure 1A. From text-mining searches, 911 genes were related to “glaucoma,” 311 genes were related to “sclera,” 519 genes were related to “mechanical strain,” 839 genes were related to “ECM remodeling,” and 88 genes were common to all the four lists (Figure 1B). Gene functional enrichment analysis in GeneCodis yielded 54 unique genes significantly associated with mechanical strain-induced scleral ECM remodeling. A PPI network that included 54 nodes with 181 edges was constructed, and 10 genes clustered in the significant gene module (Figure 1C). KEGG pathway annotation analysis for the cluster one revealed that the Hippo signaling pathway was associated with mechanical strain-induced scleral ECM remodeling (Figure 1D).

### Upregulated YAP and Collagen Type I in the OHT Model of SD Rats

**Figure 2:**
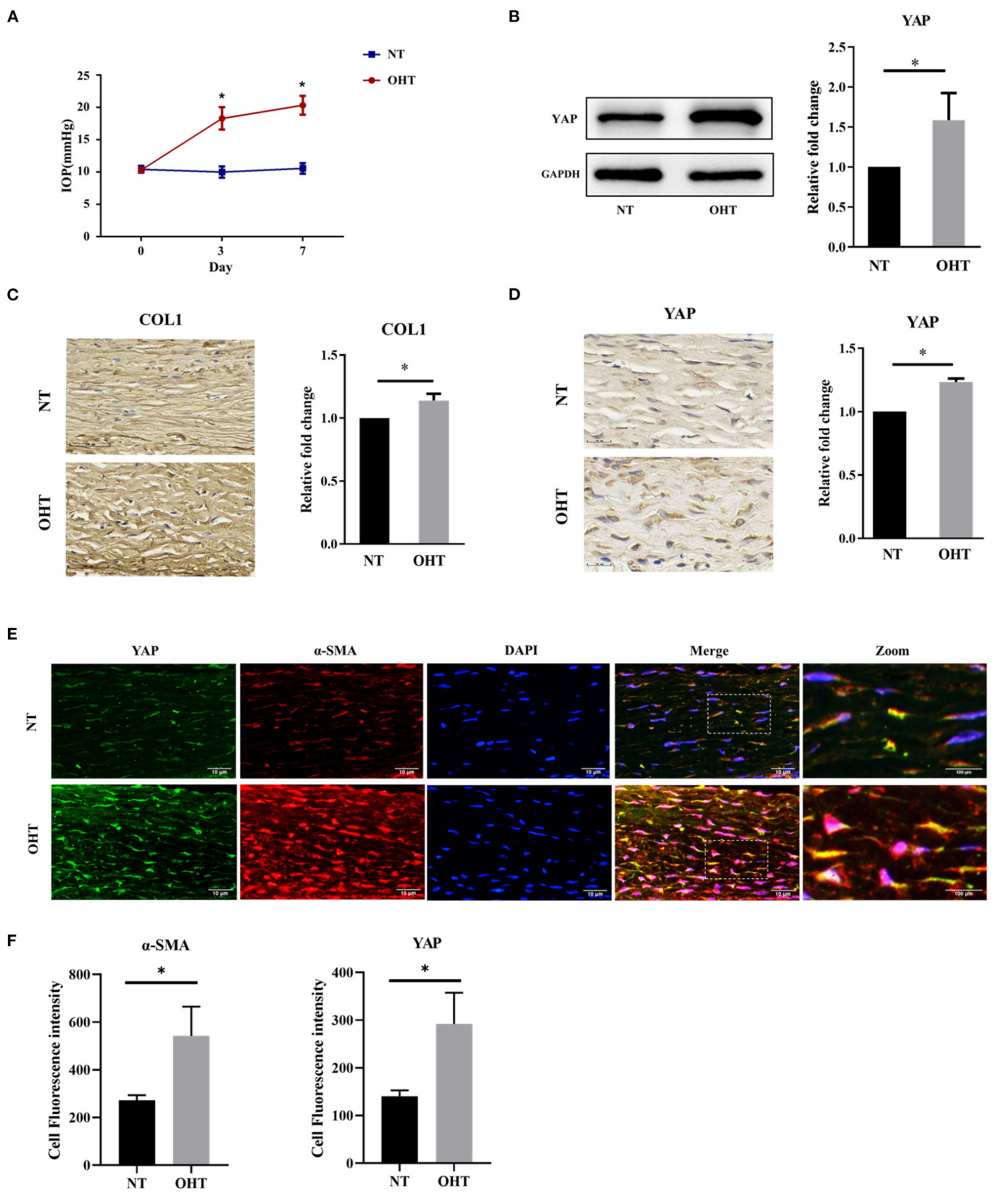
Yap expression in the OHT model of SD rats. **(A)** Intraocular pressure (IOP) in the OHT model of SD rats. **(B)** Representative picture and quantitative analysis of immunofluorescent staining of COL1 in the OHT model of SD rats (scale bar = 50 um). **(C)** Representative pictures and quantitative analysis of immunofluorescent staining of YAP in the OHT model of SD rats (scale bar = 20 um). **(D)** Western blotting analyses of YAP in the OHT model of SD rats. **(E)** Representative picture of immunofluorescence of containing YAP and α-SMA in the OHT model of SD rats (scale bar = 10 um). **(F)** Quantitative analysis of immunofluorescence of YAP and α-SMA in the OHT model of SD rats. Scleral tissues were collected after the models were successfully established for 1 week. The results are presented as means ± SD (*n* = 3), ^*^P < 0.05. Abbreviations: OHT, ocular hypertension; SD, Sprague–Dawley; NT, normotension; COL1, collagen type I; GAPDH, glyceraldehyde 3-phosphate dehydrogenase; α-SMA, α-smooth muscle actin; DAPI, 4′,6-diamidino-2-phenylindole.

First, we analyzed the expression of YAP in sclera in an OHT model of SD rats. To evaluate the effectiveness of the OHT model of SD rats used in this study, the IOP of the two eyes was measured two times a week. There were no significant differences (*P* > 0.05) in the mean IOPs between the right and left eyes of SD rats at the beginning of the experiments (Figure 2A). After injection, the mean IOPs of the injected eyes were significantly elevated compared with those of the control eyes within 1 week (all *P* < 0.001).

Collagen type I (COL1), the main component of scleral ECM, is produced by myofibroblasts, and this has been reported to be associated with fibrosis (Gentle et al., 2003). According to a previous study by our group, COL1 in sclera was initially elevated at 1 week in eyes with elevated IOP (Qiu et al., 2020). We also analyzed the expression of COL1 to ensure scleral ECM remodeling in eyes with elevated IOP. YAP is the key downstream transcriptional effector of the Hippo signaling pathway (Figure 2C) (Wada et al., 2011; Codelia et al., 2014). To determine whether YAP has a role in human scleral fibrosis, we compared the expression of YAP in the sclera between eyes with elevated IOP and the control eyes of SD rats. Immunohistochemical analysis showed that the immunoreactivity of YAP was significantly higher in eyes with elevated IOP than in control eyes (Figure 2D). Western blotting confirmed a significant increase in YAP in the sclera of eyes with elevated IOP compared to control eyes (Figure 2B).

Quantitative analysis of immunofluorescence revealed that the expression of YAP and α-SMA in sclera was significantly higher in eyes with elevated IOP than in control eyes; this reflects the Western blotting and immunohistochemistry results. Confocal microscopy data also showed YAP and colocalization of α-SMA, which revealed obvious colocalization of YAP and α-SMA in eyes with elevated IOP (Figures 2E,F). These findings support our hypothesis that enhanced YAP expression contributes to the remodeling of the sclera.

### Mechanical Strain Promotes the Expression of YAP Protein and Myofibroblast Differentiation in HSFs

To explore the effect of mechanical strain on YAP activation and myofibroblast differentiation in scleral fibroblasts, we performed Western blotting, migration assays, and CCK-8 cell proliferation assays. HSFs were subjected to 10% strain at 4, 8, 12, and 24 h. HSF cultures without applying strain at 8 h served as control groups.

**Figure 3:**
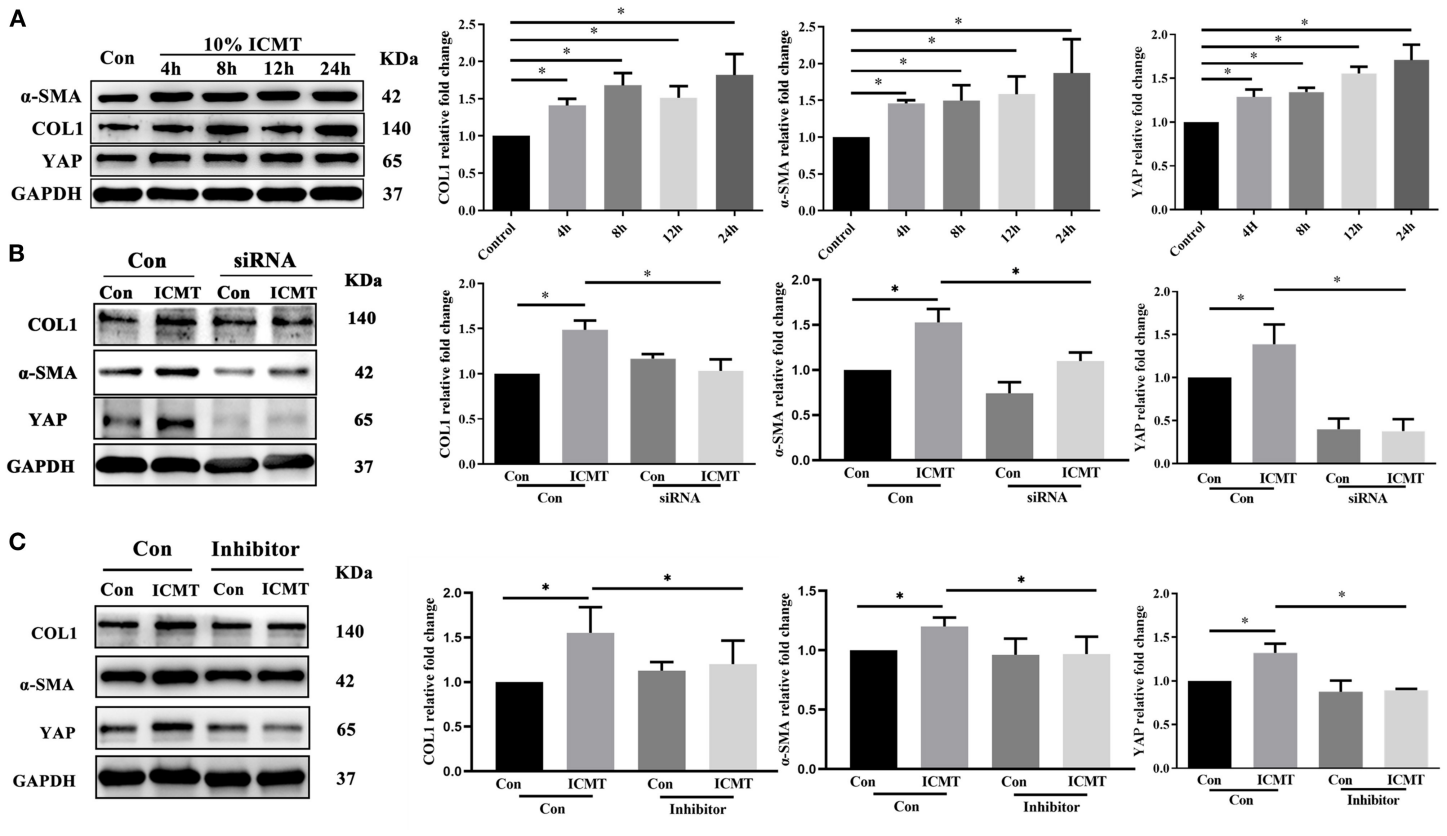
YAP is involved in the regulation of myofibroblast differentiation induced by the mechanical strain. **(A)** Western blotting and quantitative analysis of YAP, COL1, and α-SMA under mechanical strain. HSF cultures without applying strain at 8 h served as control groups. HSFs of the strain group were subjected to 10% cyclic strain at 0.5 Hz for 4, 8, 12, and 24 h. **(B)** Western blotting and quantitative analyses of YAP, COL1, and α-SMA in the YAP-specific siRNA and strain treatment groups. HSFs were transfected with the control siRNA (Con) or YAP siRNA (siRNA). HSFs of the strain group were subjected to 10% cyclic strain at 0.5 Hz for 8 h. **(C)** Western blotting and quantitative analyses of YAP, COL1, and α-SMA in the YAP-specific inhibitor and strain treatment groups. HSFs were transfected without (Con) or with verteporfin at 75 mg/ml (inhibitor). HSFs of the strain group were subjected to 10% cyclic strain at 0.5 Hz for 8 h. Data are presented as means ± SD of three separate experiments. ^**^P < 0.001. Abbreviations: Con, control; ICMT, intermittent cyclic mechanical tension; α-SMA, α-smooth muscle actin; COL1, collagen type I; GAPDH, glyceraldehyde 3-phosphate dehydrogenase.

**Figure 4:**
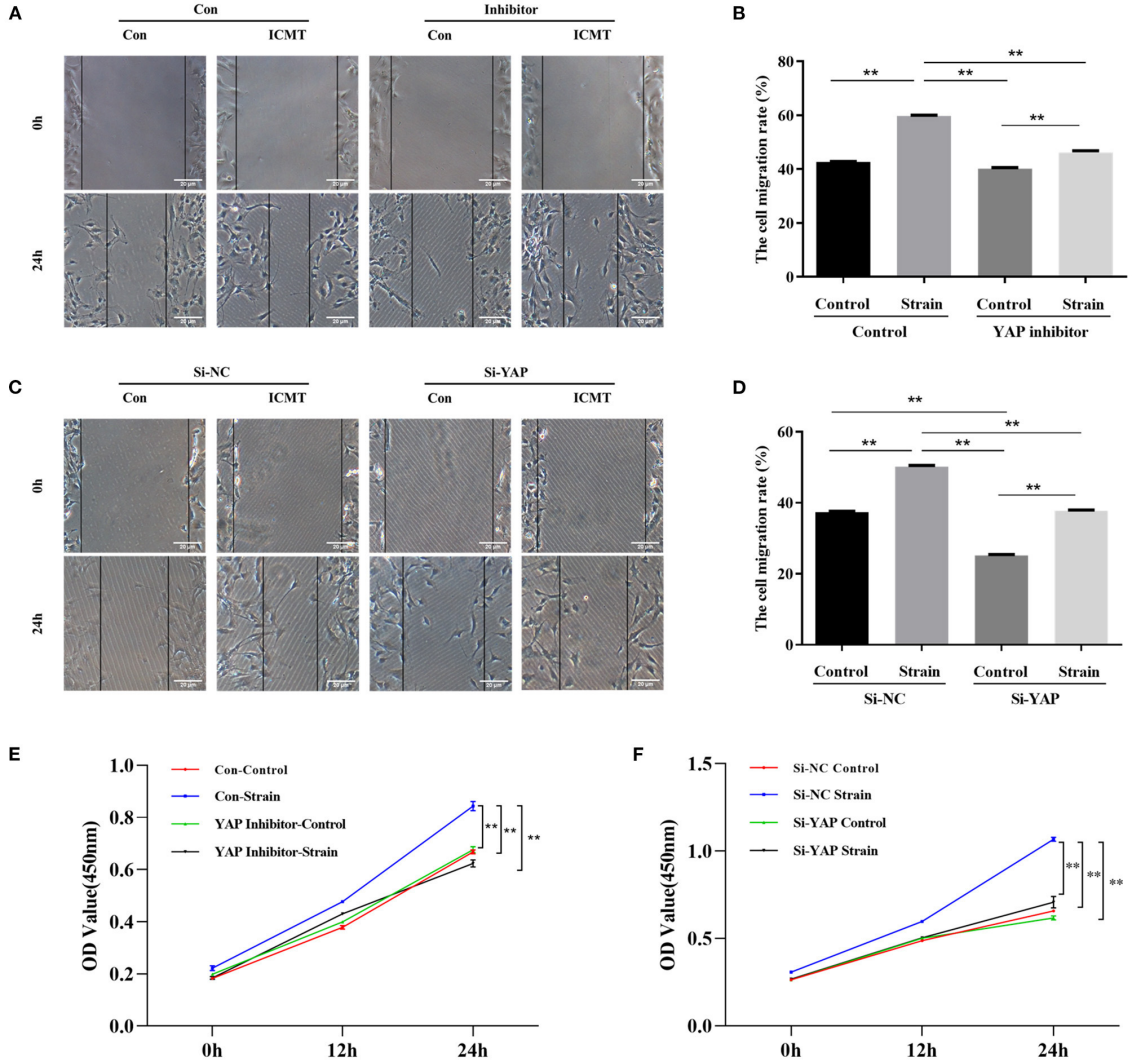
Mechanical strain induces HSFs motility and proliferation by activating YAP. **(A)** HSFs motility was detected by the scratch assay in the context of YAP inhibition. **(B)** Quantitative analysis of cell migration. **(C)** HSFs motility was detected by the scratch assay in the context of YAP knockdown. **(D)** Quantitative analysis of cell migration. **(E)** HSFs proliferative capacity was determined by CCK-8 assay in the context of YAP inhibition. **(F)** HSFs proliferative capacity was determined by CCK-8 assay in the context of YAP knockdown. HSFs of the strain group were subjected to 10% cyclic strain at 0.5 Hz for 8 h. ^**^P < 0.001. Con, control; ICMT, intermittent cyclic mechanical tension.

The protein levels of YAP in the strain group were significantly higher than those in the control group at different time points (all *P* < 0.05; Figure 3A). In addition, the protein expression levels of a-SMA and COL1 were also significantly increased in response to mechanical strain in HSFs (all *P* < 0.05). In a migration assay, HSFs subjected to mechanical strain migrated significantly faster than that of the control cells (Figure 4A, *P* < 0.05). Furthermore, the CCK-8 assay results showed that mechanical strain significantly increased the proliferation of HSFs compared with the control cells (Figures 4E,F, *P* < 0.05). These results indicated that mechanical strain induces the expression of YAP protein in HSFs and promotes the differentiation of fibroblasts into myofibroblasts.

### YAP Knockdown Downregulates Mechanical Strain-Induced a-SMA and COL1 Expression

To further assess the impact of YAP inactivation on mechanical strain-induced fibrotic changes in scleral fibroblasts, the expression of YAP was knocked down by siRNA transfection under mechanical strain conditions. Following downregulation of YAP expression, the upregulation of a-SMA and COL1 caused by mechanical strain was also significantly downregulated (Figure 3B). These results indicate that YAP is required for mechanical strain-induced myofibroblast differentiation in HSFs.

### Inhibition of YAP Suppresses Mechanical Strain-Induced Myofibroblast Differentiation

We next determined the effect of the YAP inhibitor, verteporfin, on mechanical strain-induced fibroblast to myofibroblast differentiation. Verteporfin is a porphyrinic photosensitizer clinically used for photodynamic therapy to treat age-related macular degeneration (Henney, 2000). Recent studies have shown that verteporfin could inhibit YAP activation by selectively binding YAP, altering the YAP conformation, and disrupting YAP TEAD interactions (Liu-Chittenden et al., 2012; Feng et al., 2014; Yu et al., 2014). After the application of mechanical strain, the expression of YAP protein failed to increase (*P* < 0.05, Figure 3C). Following the inhibition of YAP expression, the upregulation of a-SMA and COL1 in response to mechanical strain was also significantly downregulated. Furthermore, the increased proliferation and migration capacities induced by mechanical strain were also repressed when YAP expression was inhibited (Figures 4B–F). These results suggest that YAP plays an important role in the mechanical strain-induced myofibroblast differentiation of HSFs.

### Mechanical Strain-Induced YAP Binds to Smad3, and Inhibition of Smad3 Suppresses Myofibroblast Differentiation

**Figure 5:**
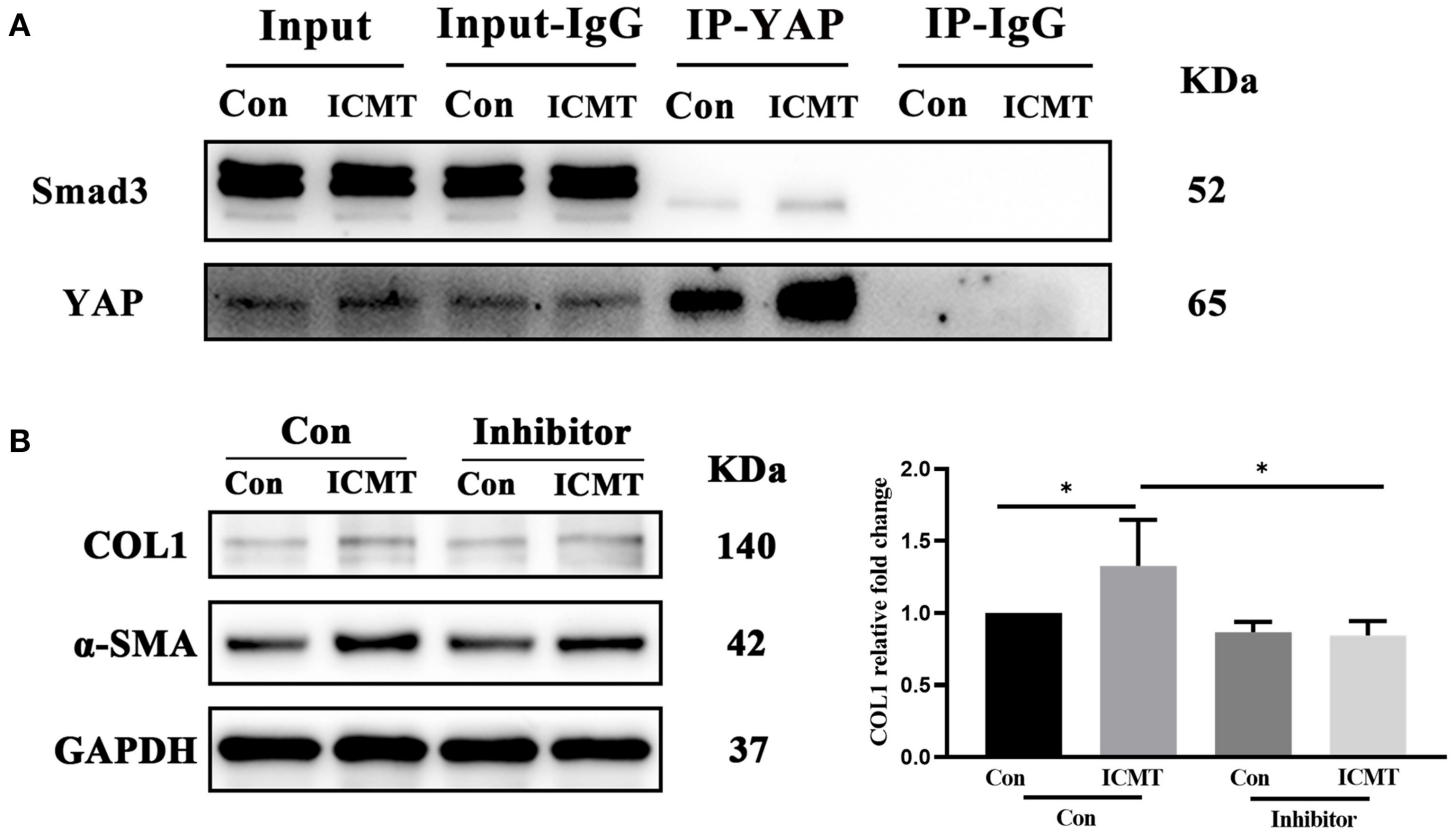
Smad3 was bound to YAP involved in myofibroblast differentiation induced by the mechanical strain. **(A)** Immunoblot of the coimmunoprecipitation was performed to test if YAP binds to Smad3. HSF cultures without applying strain at 8 h served as control groups. HSFs of the strain group were subjected to 10% cyclic strain at 0.5 Hz for 8 h. **(B)** Western blotting and quantitative analyses of COL1 and α-SMA in the Smad3-specific inhibitor and strain treatment groups. HSFs were transfected without (Con) or with SIS3 at 10 μM (inhibitor). HSFs of the strain group were subjected to 10% cyclic strain at 0.5 Hz for 8 h. Data are presented as means ± SD of three separate experiments. ^*^P < 0.05. Abbreviations: Con, control; ICMT, intermittent cyclic mechanical tension; IP, immunoprecipitation; α-SMA, α-smooth muscle actin; COL1, collagen type I; GAPDH, glyceraldehyde 3-phosphate dehydrogenase.

Smad3 is a downstream regulator of the transforming growth factor-β (TGF-β) signaling pathway, and a mediator of the fibrotic response that plays a major profibrotic role in tissue fibrosis (Flanders, 2004; Hu et al., 2018). YAP can intersect with the TGF-β pathway by forming a complex with Smad3 and thus promotes fibrosis (Grannas et al., 2015; Futakuchi et al., 2018). We next tested whether YAP was bound to Smad3. To determine whether YAP and Smad3 are physically associated with scleral fibroblasts, we performed Co-IP. As shown in Figure 5A, Smad3 was detected in the precipitated complex by YAP immunoprecipitation, and YAP was detected in the immunoprecipitated complex by Smad3 immunoprecipitation.

To further assess the role of Smad3 in mechanical strain-induced myofibroblast differentiation of HSFs, we examined the effect of inhibition of Smad3 on a-SMA and COL1 expression. SIS3, a selective inhibitor of Smad3 phosphorylation (Jinnin et al., 2006; Charbonney et al., 2011), was added to HSFs before they were subjected to mechanical strain. Western blot analysis showed that SIS3 significantly suppressed the upregulation of a-SMA and COL1 caused by mechanical strain (Figures 5B,C). Taken together, these results suggest that YAP binding to smad3 is involved in mechanical strain-induced myofibroblast differentiation of HSFs.

## Discussion

Myofibroblast differentiation of scleral fibroblasts is thought to play a critical role in mechanical strain-induced scleral ECM remodeling and in regulating the IOP-related biomechanical response of ONH (Shelton and Rada, 2007; Qu et al., 2015; Oglesby et al., 2016; Qiu et al., 2020). TGF-β2/Smad signaling (McDowell et al., 2013), Wnt/β-catenin signaling (Villarreal et al., 2014), and several biomolecular factors, such as connective tissue growth factor (Kuespert et al., 2015), MMPs, and TIMPs (Helin-Toiviainen et al., 2015), have been reported to play significant roles in the regulation of ECM remodeling in glaucoma. However, how the IOP-related strain on the sclera stimulates the differentiation of fibroblasts into myofibroblasts remains poorly understood. In this study, we demonstrated that mechanical strain induced scleral myofibroblast differentiation through Hippo-YAP signaling, and this regulation may be dependent on the proteins Smad3. We first revealed that Hippo signaling pathway may contribute to mechanical strain-induced scleral ECM remodeling through bioinformatics analysis. Furthermore, we observed that YAP was upregulated in rat scleral tissues under chronic elevated IOP for 1 week. In addition, mechanical strain induces the expression of YAP and COL1 in HSFs and promotes myofibroblast differentiation. When YAP was knocked down or inhibited, the differentiation of myofibroblasts induced by mechanical strain also was suppressed. These findings demonstrate that YAP is essential for mechanical strain-induced scleral myofibroblast differentiation.

Stiffening of the sclera resulting from ECM remodeling is one of the primary structural changes in glaucoma. The scleral stiffness of human glaucoma eyes was higher than that of normal eyes *in vivo* and *in vitro* (Hommer et al., 2008; Coudrillier et al., 2015). The stiffness of the sclera, especially the stiffness of the PPS, significantly impacts the deformation of the LC and scleral canal, influences the IOP-related biomechanical response of the ONH, and eventually affects the susceptibility to glaucomatous neuropathy (Thornton et al., 2009; Kimball et al., 2014; Coudrillier et al., 2016; Wu et al., 2020). Scleral biomechanics are determined by the composition and structure of its ECM, which is a highly dynamic complex of collagen fibrils, proteoglycans, and elastin (Boote et al., 2020). Experimental studies have shown that scleral stiffness increases after exposure to elevated IOP (Downs et al., 2005; Girard et al., 2011) and is associated with increased expression levels of fibrillin 1, COL1, and elastin in the sclera (Cone-Kimball et al., 2013; Qiu et al., 2020). In this study, we found that the protein levels of COL1 were increased in scleral tissues of eyes with elevated IOP in rats, which agreed with the previous reports.

Fibroblasts are mechanosensitive cells that are the predominant producers of ECM. Scleral fibroblasts and their ECM are the main components of the sclera and determine the IOP-related biomechanical response of the ONH which plays an important role in the pathogenesis of glaucomatous optic neuropathy (Ivers et al., 2016; Boote et al., 2020). Mechanical strain induced by IOP activates scleral fibroblast differentiation into myofibroblasts, which are responsible for ECM synthesis (Oglesby et al., 2016), secretion, and remodeling (Hinz, 2016; Bergmeier et al., 2018; DeLeon-Pennell et al., 2020). a-SMA is a marker of myofibroblasts, which is associated with the contractile activity of myofibroblasts (Tomasek et al., 2002). Increased expression of a-SMA indicates that more fibroblasts differentiated into myofibroblasts. The Hippo pathway is a critical mechanosensitive signaling pathway that is involved in cellular proliferation, survival, and differentiation (Dey and Varelas, 2020). Recently, YAP has been reported to play a crucial role in ECM remodeling by regulating myofibroblast differentiation and inducing ECM production in fibrosis of the lung, kidney, liver, and heart (Kim et al., 2019). Our bioinformatics analysis, in agreement with previous studies, showed that the Hippo signaling pathway was a key pathway of mechanical strain-induced scleral ECM remodeling in glaucoma. Furthermore, the *in vivo* study showed an increase in YAP and a-SMA expression in the hypertensive sclera of SD rats. Increased YAP may be involved in scleral myofibroblast differentiation and ECM remodeling.

To investigate the role of YAP in scleral myofibroblast differentiation in glaucoma, a mechanical stress-stretch model was used *in vitro* to mimic IOP-induced mechanical strain. Increased expression of YAP was observed in HSFs after 10% strain application for 4–24 h. This increase in YAP coincided with increased expression of a-SMA and COL1 and increased proliferation and migration capacities of scleral fibroblast cells. This is consistent with our previous study showing that mechanical strain can enhance the proliferation and migration capacities of scleral fibroblast cells, and increase the expression of a-SMA in scleral fibroblast cells (Qiu et al., 2018). When YAP expression was knocked down or inhibited, the upregulation of a-SMA and COL1 induction by mechanical strain was significantly suspended. Furthermore, the increased proliferation and migration capacities induced by mechanical strain were also repressed when YAP expression was inhibited. This suggested that YAP regulated mechanical strain-induced scleral myofibroblast differentiation and ECM remodeling of the sclera. The Hippo pathway effector YAP is a primary sensor of the physical nature of the cell that mediates cellular responses to the mechanical strain (Codelia et al., 2014). Previous studies have reported that YAP is a key regulator of fibroblast activation that has been implicated in pathological fibrosis of the lung and renal fibrosis. Liu et al. (2015) previously showed that YAP plays an important role in pulmonary fibrosis. Szeto et al. (2016) reported that YAP was involved in myofibroblast differentiation induced by ECM stiffness and renal fibrogenesis. Recent studies have indicated that YAP regulates TGF-β1-mediated myofibroblast differentiation and ECM remodeling in conjunctival fibrosis (Futakuchi et al., 2018), which is consistent with our study.

In this study, the expression of a-SMA increased from 4 h of 10% strain. In the study by Meng et al. (2010), human periodontal ligament fibroblasts were subjected to 0.5 Hz cyclic mechanical tension and the expression of a-SMA increased from 3 h, which was consistent with this study. In the study by Yuan et al. (2018), pig scleral fibroblasts were subjected to 4% cyclic strain for 24 h and observed a significant increase in a-SMA expression. However, they did not investigate the change of a-SMA expression before 24 h. The previous study also indicated that the protein expression of fibroblasts, like SMA and fibronectin, would change from 4 to 12 h of various stimuli (Weis et al., 2013; Gao et al., 2014). Differential time of protein expression in fibroblasts might be related to many factors, such as cell type and strength of the stimulus.

Smad3 is a key mediator of fibrogenesis that plays a major profibrotic role in tissue fibrosis (Flanders, 2004; Hu et al., 2018). Previous studies have reported that YAP can intersect with the TGF-β pathway by forming a complex with Smad3 and thus regulate fibrotic processes (Varelas et al., 2010; Grannas et al., 2015; Futakuchi et al., 2018). During the liver fibrosis process, the expression of YAP and Smad3 tended to increase with the worsening of the disease (Chen et al., 2018). Szeto et al. (2016) reported that YAP/TAZ cooperates with Smad2/3 to control mechanical strain-induced renal fibrogenesis. Recent evidence indicates that YAP directly regulates TGF-b2-induced conjunctival fibrosis by modulating the Smad2/3 pathway (Futakuchi et al., 2018). In the current study, we verified that YAP interacted with Smad3 by a Co-IP assay and that inhibition of Smad3 suppressed scleral myofibroblast differentiation induction by a mechanical strain. This finding indicated that YAP maybe regulates scleral myofibroblast differentiation by binding to Smad3.

## Conclusion

In conclusion, this study shows that YAP plays a critical role in mechanical strain-induced fibrosis in HSFs. Mechanistic strain upregulated the expression of YAP, which subsequently promoted the differentiation of fibroblasts into myofibroblasts and mediated scleral ECM remodeling. This study is a preliminary study of the role of YAP in the mechanical strain-induced scleral myofibroblast transformation through *in vitro* model. Further *in vivo* studies are required to determine the role of YAP in scleral fibrosis during glaucoma.

## Data Availability Statement

The original contributions presented in the study are included in the article, further inquiries can be directed to the corresponding author.

## Ethics Statement

The animal study was reviewed and approved by Institutional Review Board and Ethics Committee of Eye and Ear, Nose, Throat Hospital of Fudan University.

## Author Contributions

DH, XS, and SQ conceived the study. DH, BD, and JJ performed the experiments. DH and BD analyzed the data. DH and JJ wrote the paper. DH, KX, and SQ revised the manuscript. All authors contributed to the article and approved the submitted version.

## Funding

The authors were supported by grants from the Natural Science Foundation of Shanghai (18ZR1406000), the State Key Program of National Natural Science Foundation of China (no. 81430007), the National Natural Science Foundation of China (no. 81790641), and the top priority of Clinical Medicine Center of Shanghai (2017ZZ01020). The project was also supported by the Shanghai Committee of Science and Technology, China (Grant No. 20Y11911200). The sponsor or funding organization had no role in the design or conduct of this research.

## Conflict of Interest

The authors declare that the research was conducted in the absence of any commercial or financial relationships that could be construed as a potential conflict of interest.

## Publisher's Note

All claims expressed in this article are solely those of the authors and do not necessarily represent those of their affiliated organizations, or those of the publisher, the editors and the reviewers. Any product that may be evaluated in this article, or claim that may be made by its manufacturer, is not guaranteed or endorsed by the publisher.
